# Symbiotic T6SS affects horizontal transmission of *Paraburkholderia bonniea* among *Dictyostelium discoideum* amoeba hosts

**DOI:** 10.1093/ismeco/ycaf005

**Published:** 2025-01-14

**Authors:** Anna Chen, Rachel M Covitz, Abigail A Folsom, Xiangxi Mu, Ronald F Peck, Suegene Noh

**Affiliations:** Biology Department, Colby College, 5717 Mayflower Hill, Waterville, ME 04901, United States; Biology Department, Colby College, 5717 Mayflower Hill, Waterville, ME 04901, United States; School of Medicine, University of Pittsburgh, 3550 Terrace Street, Pittsburgh, PA 15213, United States; Biology Department, Colby College, 5717 Mayflower Hill, Waterville, ME 04901, United States; Biology Department, Colby College, 5717 Mayflower Hill, Waterville, ME 04901, United States; Biology Department, Colby College, 5717 Mayflower Hill, Waterville, ME 04901, United States; Biology Department, Colby College, 5717 Mayflower Hill, Waterville, ME 04901, United States

**Keywords:** secretion system, horizontal transmission, host fitness, symbiont fitness, flow cytometry, protist, virulence factor

## Abstract

Three species of *Paraburkholderia* are able to form facultative symbiotic relationships with the amoeba, *Dictyostelium discoideum*. These symbiotic *Paraburkholderia* share a type VI secretion system (T6SS) that is absent in other close relatives. We tested the phenotypic and transcriptional effect of *tssH* ATPase gene disruption in *P. bonniea* on its symbiosis with *D. discoideum*. We hypothesized that the ∆*tssH* mutant would have a significantly reduced ability to affect host fitness or transmit itself from host to host. We found that the T6SS does not directly affect host fitness. Instead, wildtype *P. bonniea* had significantly higher rates of horizontal transmission compared to ∆*tssH*. In addition, we observed significant differences in the range of infection prevalence achieved by wildtype vs. ∆*tssH* symbionts over multiple host social stages in the absence of opportunities for environmental symbiont acquisition. Successful symbiont transmission significantly contributes to sustained symbiotic association. Therefore, the shared T6SS appears necessary for a long-term evolutionary relationship between *D. discoideum* and its *Paraburkholderia* symbionts. The lack of difference in host fitness outcomes was confirmed by indistinguishable host gene expression patterns between hosts infected by wildtype or ∆*tssH P. bonniea* in an RNA-seq time series. These data also provided insight into how *Paraburkholderia* symbionts may evade phagocytosis by its amoeba host. Most significantly, cellular oxidant detoxification and lysosomal hydrolase delivery appear to be subject to the push and pull of host-symbiont crosstalk.

## Introduction

Bacterial secretion systems, particularly types III, IV, and VI, are critical virulence determinants in several Gram-negative pathogens [1–4]. In the context of host-pathogen interactions, they can be used by bacteria to deliver a variety of effector molecules directly into eukaryotic host cells. We sought to determine the role of a shared type VI secretion system (T6SS) in enabling a persistent symbiotic relationship between *Dictyostelium discoideum* amoebas and their *Paraburkholderia* symbionts. *D. discoideum* is a soil-dwelling predator of bacteria that has also proven useful for modeling host-pathogen interactions in the lab [5–7]. *D. discoideum* interacts with and attempts to eliminate internal pathogens through phagocytosis [8, 9]. But three species of *Paraburkholderia*, *P. agricolaris, P. hayleyella,* and *P. bonniea* are able to avoid digestion and form facultative symbiotic relationships with *D. discoideum* [10, 11]. Approximately a third of wild isolates of *D. discoideum* have been stably associated with these symbiotic *Paraburkholderia* [12, 13].

*D. discoideum* has a unique life cycle [14, 15]. Single cell vegetative amoebas enter their social stage in response to starvation. Amoebas aggregate and form a multicellular slug. The slug migrates, then differentiates into a fruiting body made up of a stalk and a sorus. The amoebas that survive the social stage become spores contained in the sorus. These spores then disperse and germinate into vegetative amoebas. During both the single cell and social stages of their life cycle, infected *D. discoideum* persistently carry their symbiotic *Paraburkholderia* [12, 16, 17]. The nature of the symbiotic relationship is actively under study, and association outcomes of *D. discoideum* hosts differ depending on the symbiont species and strain [16, 17].

The three symbiotic *Paraburkholderia* species form two distant clades, yet share a type III secretion system (T3SS) and type VI secretion system (T6SS) that are absent in other close relatives [18]. We hypothesized that the ability of *Paraburkholderia* to persistently infect *D. discoideum* may be partially attributed to the shared T3SS and T6SS*.* The shared T6SS phylogenetically clusters with T6SS-5 of the pathogenic *Burkholderia* clade consisting of *Burkholderia pseudomallei*, *B. mallei*, *B. thailandensis*, and *B. oklahomensis*. T6SS-5 has been shown to be functionally linked to virulence in these opportunistic mammalian pathogens [19–21]. Hosts of *B. pseudomallei* are subject to multinucleated giant cells formation as a result of cell-to-cell fusion [22], and pathogen transmission occurs across these giant cells via T6SS-5 [23, 24].

The cellular machinery of the T6SS consists of three main complexes: the membrane complex that docks the remainder of the secretion system to the bacterial cell envelope, the baseplate complex that anchors the tail complex to the membrane complex, and the contractile sheath tail complex that is topped by the spike complex comprising VgrG and PAAR repeat proteins [3, 25]. Components of the T6SS are evolutionarily related to contractile bacteriophage tails, and contractile tail elongation through Hcp protein polymerization propels effectors across membranes [26]. The TssH ATPase (also known as ClpV) is recruited to the contracted sheath to recycle the sheath and other T6SS components for repeated firing [27]. In *B. pseudomallei*, *tssH* is one of the macrophage-inducible genes that contributes to virulence [28].

We tested the phenotypic and transcriptional effect of *P. bonniea tssH* ATPase gene disruption on their symbiosis with *D. discoideum*. We hypothesized that the *P. bonniea* ∆*tssH* ATPase mutant would have a significantly reduced ability to influence host fitness or transmit itself from host to host compared to the wildtype due to reduced delivery of effector proteins. We also generated RNA-sequencing data to understand the molecular impact of type VI secretion perturbation on the host in this amoeba-bacteria symbiosis.

## Materials and methods

### Bacteria and amoeba samples

We cultured *P. bonniea* and *Klebsiella pneumoniae* food bacteria on SM/5 plates [2 g glucose, 2 g BactoPeptone (Oxoid), 2 g yeast extract (Oxoid), 0.2 g MgCl_2_, 1.9 g KH_2_PO_4_, 1 g K_2_HPO_4_, and 15 g agar per liter]. Two *D. discoideum* host strains not associated with *Paraburkholderia* in the wild were used for all experiments as biological replicates (QS4, QS864). Both strains were originally isolated from Mt. Lake Biological Station in Virginia, USA. We used two pairs of red fluorescent protein (RFP) labeled *P. bonniea* strains (bb433, bb859) [17]. We had previously found that bb433 and bb859 are genetically distinct and cause significantly different phenotypes when associated with *D. discoideum* [29]. We grew *D. discoideum* on SM/5 plates with 2 × 10^5^ spores and *K. pneumoniae* (200 μl at 1.5 OD_600 nm_) in a 21°C incubator for all assays. We grew all bacteria at room temperature. We used KK2 buffer (2.2 g KH_2_PO_4_ monobasic and 0.7 g K_2_HPO_4_ dibasic per liter) for handling bacteria, *D. discoideum* spores, and amoebas for all assays.

### tssH mutant generation

Mutant *P. bonniea* were generated with Gateway-compatible allelic exchange using homologous recombination in *P. bonniea* strain bb433 and bb859 backgrounds. A two-step polymerase chain reaction (PCR) was performed to construct a recombinant plasmid to allow an in-frame deletion of codons 8–957 (2850 bp) of the 966-codon open reading frame. Primers were designed using Geneious Prime version 2020.2.5 (https://www.geneious.com) using the *P. bonniea* strain bb859 genome as template. In the first step, PCR reactions were carried out with primer pairs (lowercase letters bind to genomic DNA template): RP966 (ACAAAAAAGCAGGCTgggcaacgatatcgagcagatc) with RP967 (TCCGGTCGCTTTTGTGGCcttgaggtcgagctgaatcatg), and RP968 (gccacaaaagcgaccgga) with RP969 (TACAAGAAAGCTGGGTtgtcgaagtgctcgccatag). These PCR products were used as templates in the second-step PCR with primers to generate the gene deletion and add *attB1* and *attB2* sites: RP956 (GGGGACAAGTTTGTACAAAAAAGCAGGCT) and RP957 (GGGGACCACTTTGTACAAGAAAGCTGGGT). This product was then used in a Gateway cloning reaction with pDONRPEX18Tp-SceI-pheS [30] to generate plasmid pRFP505 which was introduced into *P*. *bonniea* by electroporation. Genomic integrants were confirmed by PCR, and then transformed with pDAI-SceI-pheS [30] and recombinants were screened by PCR again. To verify successful deletion of the *tssH* ATPase, Oxford Nanopore sequencing was performed at Seqcenter (Pittsburgh, PA). A BLAST library of all sequenced reads was constructed using BLAST v2.14.0+ [31]. Within the library, we queried for the deleted gene. Successful deletion was indicated by a lack of matches to the full nucleotide sequence of tssH, and instead the 5′ and 3′ sequences of *tssH* were joined together.

### tssH rescue generation

To generate a plasmid for expression of *tssH*, primers RP1046 (GAGCTTCGAAAGGACAAGCATA*atg*attcagctcgacctcaagg) and RP1047 (TCATGTTTGACAGCTTACTCGA*tca*tggagttactccggtcg) (lower case letters bind to genomic DNA template, start and stop codons italicized) were used in PCR with the *P. bonniea* strain bb859 genome as template to amplify *tssH*. pDAI-SceI-pheS [30] was cut with the restriction enzymes *Nde*I and *Xho*I and the 7 kb fragment was isolated. New England Biolabs (Ipswich, MA) HiFi DNA assembly was used to combine the plasmid fragment with the PCR product. The resulting plasmid (pRFP533) allows constitutive expression of *P. bonniea tssH* driven by the P*_S12_* promoter of the *B. pseudomallei rpsL* gene [32]. The plasmid sequence was confirmed by whole plasmid sequencing by Plasmidsaurus (San Francisco, CA) using Oxford Nanopore Technology. pRFP533 was introduced into P. bonniea ∆*tssH* mutants by electroporation and the presence of the plasmid was confirmed by PCR.

### Host fitness

To measure any differences in host fitness due to association with *P. bonniea* wildtype vs. ∆*tssH*, we estimated host fitness as spore production across a range of infection prevalence [29]. Triplicate *D. discoideum* associations were prepared at the following multiplicities of infection (MOI) along with food bacteria: uninfected (control), 0.3, 1.5, and 7.5 MOI of *P. bonniea* cells to *D. discoideum* spores. For each *P. bonniea* strain wildtype and ∆*tssH* pair, we conducted the experiment with two different host strains as biological replicates. We incubated the newly infected *D. discoideum* cultures for 5–7 days until they formed fruiting bodies. Spores were then harvested for infection prevalence and spore count assays. Infection prevalence was defined as the percent of RFP+ infected spores in a sample of 100 000 cells analyzed on a Sony Biotechnology SH800 flow cytometer (San Jose, CA). The resulting FCS files were imported into FlowJo v10.8.1 for analysis [33] where we first gated *D. discoideum* spores, then determined RFP+ cells from the RFP- cells using the negative control and highest MOI samples as gating references. Total spore counts were quantified using a 200x dilution sample with a hemocytometer.

### Symbiont transmission

To measure symbiont horizontal transmission, we exposed pre-infected amoebas to uninfected amoebas and estimated horizontal transmission as the percentage of newly infected spores in fruiting bodies after completion of a social stage [29]. For each *P. bonniea* strain wildtype and ∆*tssH* pair, we conducted the experiment with two different host strains as biological replicates. The pre-infected *D. discoideum* were prepared as above. After fruiting bodies formed, we collected the infected spores. The infected spores and uninfected spores were separately plated onto fresh SM/5 plates at densities of 2 × 10^5^ and 1 × 10^5^ spores per plate with food bacteria. 36 hours post-incubation, amoebae in log-phase growth were collected. The uninfected amoebas were dyed with CellTracker™ green CMFDA (Invitrogen) dissolved in DMSO. Pre-infected amoebas carrying RFP+ *P. bonniea* were exposed to DMSO only. Both sets of amoebas were then washed and processed in parallel. Next, the uninfected dyed amoebas were combined with the infected RFP+ amoebas at 1:0 (dye-only control), 0:1 (infected only control), and 1:1 (mixed) ratios. These mixes were spread onto nitrocellulose filters (Millipore) moistened with KK2 in replicates of three and left to develop into fruiting bodies in the dark. We collected spores 5–7 days later and analyzed samples on a SH800 flow cytometer. In FlowJo v10.8.1, spores were gated, then determined gates for dyed cells from the dye-only control, and infected cells from the highest MOI sample on their respective channels. Horizontal transmission was detected by measuring the percent of spores in which the green-labeled dye signal and red-labeled infection signal co-occurred. The spores in this population were previously uninfected amoebas but that were now RFP+ for symbiont presence.

### Statistical analysis

Statistical analyses were performed using R v.4.4.1 with packages car v.3.1–2 [34] and lme4 v.1.1–35 [35]. For both host fitness and symbiont transmission, we fit linear models with mixed effects. Infection prevalence from flow cytometry data was coded as a continuous predictor, and the linear model accounted for experiment date and host strain as random effects. We tested how host fitness (relative percent of spores produced) was affected by symbiont treatment (wildtype or ∆*tssH*), and how symbiont transmission was affected by symbiont treatment. Residuals were evaluated on the final model to ensure model assumptions were met. For both host fitness and symbiont transmission, we used the package emmeans v.1.10.3 [36] and its emtrends() function for post-hoc tests of significant differences between pairwise slopes by treatment within symbiont background, and emmeans() for post-hoc tests of significantly different means by treatment within symbiont background. *P*-values were adjusted using Tukey’s method.

### RNA-sequencing

We aimed to detect gene expression changes between hosts upon infection with *P. bonniea* wildtype vs. ∆*tssH* mutants. We infected two *D. discoideum* strains, QS4 and QS864 with *P. bonniea* bb433 wildtype or *∆tssH*, and collected cells at 90, 360, and 2250 minutes post-infection (MPI). To conduct the time series, two days prior to the experiment day, we prepared uninfected and infected *D. discoideum* samples. The infected samples modeled longer-term infections (reaching ~2250 MPI at time of collection) and were prepared at MOI that led to similar infection prevalence in pilot experiments (wildtype at 24 MOI or ∆*tssH* at 60 MOI). One day prior to the experiment, *P. bonniea* plates were prepared. The next day, both amoebae and *P. bonniea* were collected in log-phase growth. With these cells, we prepared replicates of uninfected and infected amoebas, new wildtype infections (at MOI 24), and new ∆*tssH* infections (at MOI 60) by depositing samples onto plates containing food bacteria grown overnight. Negative controls, 90, and 2250 MPI plates were collected for both wildtype and ∆*tssH* infections at 90 minutes thereafter. Additional 360 MPI plates for both wildtype and ∆*tssH* infections were collected at 360 minutes thereafter. All cells were collected into RNAlater (Qiagen). Subsequent total RNA extractions from each sample were performed with RNeasy plus Mini kits (Qiagen). Illumina stranded mRNA-seq libraries were prepared and sequenced as 150 base pair paired-end reads at University of Minnesota Genomics Center (Minneapolis, MN). Libraries were barcoded, pooled, and sequenced across three lanes to minimize lane (batch) effects.

### RNA-seq analysis

We performed quality control on the sequencing reads using fastp v.0.23.1 [37]. Reads at least 20 bp long and with at least ⅓ of each bases with PHRED base quality scores above 30 were retained. Reference data was obtained from DictyBase [38, 39] on January 16, 2023, and included a *D. discoideum* reference genome (FASTA), a gene annotation file (GTF), and a Gene Ontology (GO) annotation file. The GTF file was used by STAR to identify splice junctions for alignment. The reference genome was indexed and reads were aligned to the indexed reference using STAR v.2.7.5c [40]. Only reads mapped in proper pairs were retained and sorted using SAMtools v.1.3.1 [41]. Read groups were also added using SAMtools. Count tables were generated using htseq-count v.0.11.2 [42] with the -s reverse flag and minimum alignment quality score threshold of 20.

Differential expression analysis was performed with DESeq2 v.1.44.0 [43] and visualization with ggplot2 v.3.5.1 [44], RColorBrewer v.1.1–3 [45], and gridExtra v2.3 [46]. We performed two analyses. First, a pairwise contrast of differential gene expression between the wildtype vs. ∆*tssH* mutant was performed. Then, to understand how symbiotic association develops, we made three pairwise contrasts for each timepoint compared to the negative control. For each contrast, differential expression analysis was performed with a false discovery rate of 0.05. Venn diagrams were generated using limma v.3.60.4 [47]. Clusters of phagocytosis genes with similar expression changes over time were detected using DEGreport v.1.40.1 [48].

### GO term enrichment analysis

GO enrichment analysis was performed on the identified candidate genes from DESeq2 using GOstats v.2.70.0 [49]. To prepare for GO term enrichment, annotations with “NOT” qualifiers evidence codes, “ND” (No biological Data available) evidence codes, and annotations lacking a source were filtered out. Lists of up- and down-regulated genes were constructed. We used GSEABase v.1.66.0 to load the respective gene list, ontology file, and GO annotations [50]. We used GOstats to perform a hypergeometric significance test with a p-value cut off of 0.01 to test for enrichment using counts in up and downregulated genes. To visualize the GO term enrichment results, semantic bubble plots were generated with GO-Figure v.1.0.1 [51] using GOstats result tables.

## Results

### Host fitness

**Figure 1:**
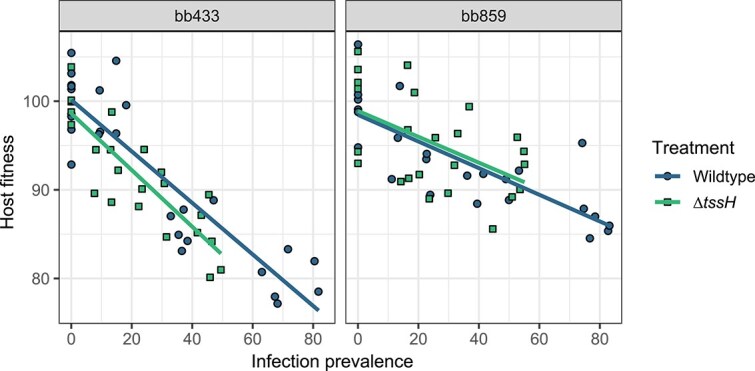
Host fitness was negatively correlated with infection prevalence after a single post-infection social stage. Infection prevalence was estimated per sample as the percent of infected host spores positive for RFP-labeled symbionts. Host fitness was estimated by the percent of spores produced per sample relative to uninfected hosts prepared at the same. The host fitness slopes were not significantly different between wildtype and ∆*tssH* within each symbiont background. As previously observed (Noh et al 2024), bb433 strains more negatively impact host fitness compared to bb859 strains.

**Figure 2:**
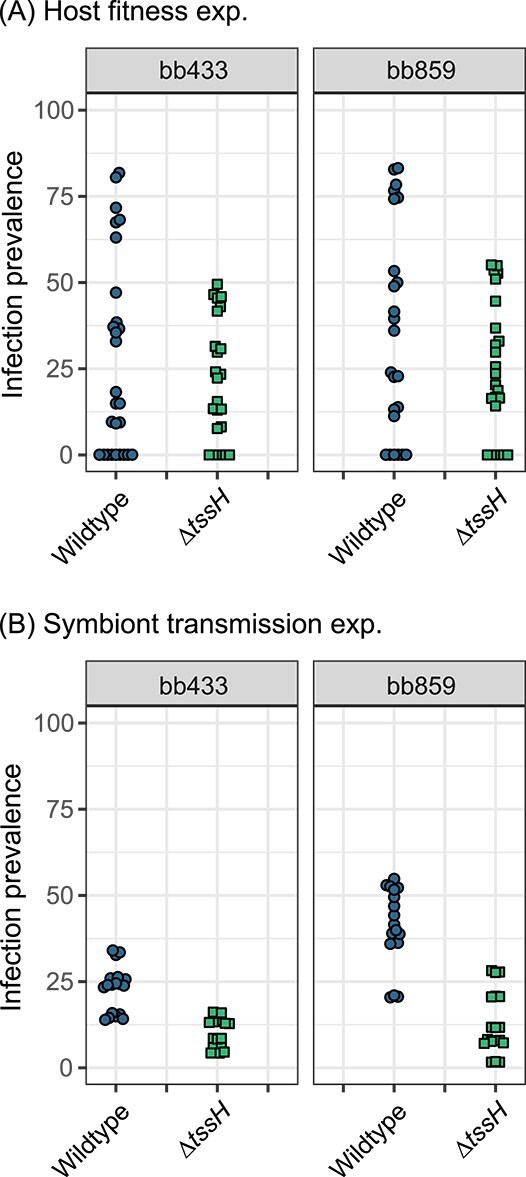
Infection prevalence was lower in ∆*tssH P. bonniea* compared to wildtype. The difference is exacerbated when amoebas are observed at additional social stages after infection: The host fitness experiment (A) incorporates one social stage while the symbiont horizontal transmission experiment (B) incorporates two social stages.

Host fitness was measured as relative spore counts produced by infected *D. discoideum* hosts against uninfected controls. As previously observed [29], overall host fitness decreased as infection prevalence increased for all combinations of host and symbiont (*χ*^2^ = 185.175, *P* < .001; Fig. 1), and the two *P. bonniea* backgrounds resulted in different slopes (*χ*^2^ = 21.032, *P* < .001; Table 1). However, we found that the host fitness response slope was not significantly different between wildtype and *∆tssH* infected hosts (*χ*^2^ = 0.088, *P* = .767). Although not significant, the mean infection prevalence trended lower for the ∆*tssH* mutant across both symbiont backgrounds (bb433: wildtype-∆*tssH =* 6.23, *P* = .443; bb859: wildtype-∆*tssH =* 10.47, *P* = .455; Fig. 2a)*.*

**Table 1:** Analysis of Deviance table for host fitness in *D. discoideum* exposed to wildtype vs. ∆*tssH P. bonniea* mutants.

Variable	*X* ^2^	*df*	*P-value*	${\bf\eta}_{\bf{\textit p}}^{\bf{2}}$	*95% CI*
Infection prevalence	185.175	1	< 0.001	0.62	[0.52, 1]
Symbiont background	18.719	1	< 0.001	0	[0, 1]
Symbiont treatment	1.167	1	0.280	0	[0, 1]
Infection prevalence: background	21.032	1	< 0.001	0.18	[0.07, 1]
Infection prevalence: treatment	0.088	1	0.767	0	[0, 1]
Background: treatment	3.003	1	0.083	0.02	[0, 1]

### Symbiont transmission

**Figure 3:**
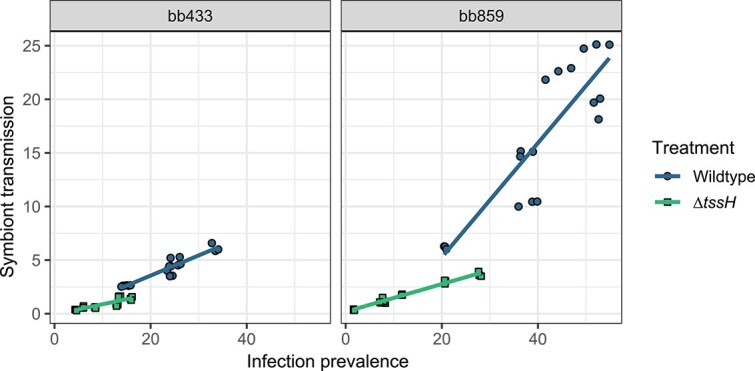
The rate of symbiont horizontal transmission was positively correlated with pre-infection prevalence after two post-infection social stages. Infection prevalence was estimated per sample as above. Horizontal transmission was estimated by the percent of RFP+ infected host spores in the test sample that were also positive for a membrane dye. The dyed cells were uninfected prior to the experiment, during which they were combined with pre-infected (and undyed) cells. The horizontal transmission rate slopes were significantly different between wildtype vs. ∆*tssH* mutants for bb859, but not bb433. Across both symbiont backgrounds, mean infection prevalence was also significantly different between wildtype vs. ∆*tssH* mutants. As previously observed (Noh et al 2024), wildtype bb433 has a lower rate of horizontal transmission compared to wildtype bb859.

We estimated symbiont horizontal transmission by the percentage of newly infected spores from previously uninfected *D. discoideum* hosts resulting from interactions with hosts pre-infected with *P. bonniea*. As previously observed [29], overall horizontal transmission increased with infection prevalence of pre-infected amoebas (*χ*^2^ = 171.780, *P* < .001; Fig. 3). However, the rate of horizontal transmission was affected by both *P. bonniea* background (*χ*^2^ = 13.458, *P* < .001) and symbiont treatment (*χ*^2^ = 114.727, *P* < .001) (Table 2). Across both backgrounds, mean infection prevalence was lower for ∆*tssH* mutants compared to wildtypes (bb433: wildtype-∆*tssH =* 12.4, *P* = .002; bb859: wildtype-∆*tssH =* 27.9, *P* < .001; Fig. 2b). For bb859, but not bb433, ∆*tssH* mutants had shallower slopes of horizontal transmission compared to their respective wildtype counterparts (bb433: wildtype-∆*tssH* = 0.111, *P* = .216; bb859: wildtype-∆*tssH* = 0.523, *P* < .001; Fig. 3). Finally, there was a significant three-way interaction by symbiont background and treatment that is likely the result of lower infection prevalence observed in bb433 relative to bb859 (*χ*^2^ = 17.267, *P* < .001).

When *tssH* expression plasmids were added to ∆*tssH* mutants (p*tssH*), these symbionts were able to recover wildtype levels of horizontal transmission (Supplemental Fig. S1, Table 3). The slopes for wildtype vs. p*tssH* were not significantly different from each other (bb433: wildtype-p*tssH* = −0.050, *P* = .212; bb859; wildtype-p*tssH* = −0.050, *P* = .212) and the mean infection prevalence of wildtype vs. p*tssH* were not significantly different from each other (bb433: wildtype-p*tssH* = 1.52, *P* = .993; bb859; wildtype-p*tssH* = 3.42, *P* = .967).

### RNA-seq time series

Two host strains infected with wildtype vs. ∆*tssH P. bonniea* bb433 showed no difference in gene expression during early stages of association at 90, 360, and 2250 MPI. Out of 8490 expressed genes, none were significantly differentially expressed (FDR = 0.05). This was consistent with the results of host fitness assays, which failed to find a significant difference between host responses to wildtype and ∆*tssH* (Fig. 1).

Despite the lack of difference in host response to infection with wildtype and ∆*tssH* mutants, the time series data offered an opportunity to understand how hosts respond to association with *P. bonniea*. Therefore we compared differential expression across host strains and symbiont treatments at each timepoint relative to the uninfected negative control (FDR = 0.05). Overall patterns of transcription indicated a sharp departure of the 360 MPI timepoint from all other time samples (Fig. 5), which was also reflected in the greater number of differentially expressed genes. Out of 8490 expressed genes, 82 (0.97%) were up- and 129 (1.5%) were down-regulated at 90 MPI, 1519 (18%) were up- and 1532 (18%) were down-regulated at 360 MPI, and 630 (7.4%) were up- and 772 (9.1%) were down-regulated at 2250 MPI (Supplemental Fig. S2). Both up- and down-regulated differentially expressed genes only share ~1% of genes among all timepoints as infection progresses, but 10% of up-regulated genes and 14% of down-regulated genes overlapped between 360 and 2250 MPI (Supplemental Fig. S3). These results suggest 360 MPI as being closest to the peak of a host’s response to infection with *P. bonniea* among our timepoints and that a sustained host response follows afterward.

We used both GO enrichment tests and semantic analysis of enriched GO terms at each timepoint (Supplemental Fig. S4, Supplemental Tables S1–S6). At 90 MPI, the up-regulated functions in our infected host cells included potassium ion transport, glutamine metabolism, and cellular responses to nutrient stress. The down-regulated functions at 90 MPI included various amino acid, fatty acid, and organic acid metabolic processes, antibiotic metabolic process, sphingolipid metabolic process, and oxidation–reduction process. No distinct clusters of GO terms were seen in the semantic analysis due to the relatively low number of differentially expressed genes.

At 360 MPI, major up-regulated semantic clusters encompass amino acid metabolism, tricarboxylic acid metabolism, nucleoside and nucleotide metabolism, oxidative phosphorylation, respiratory electron transport chain, and phagocytosis and several related terms (actin filament organization and polymerization, vesicle-mediated transport, and proton transmembrane transport). The enrichment of phagocytosis-related terms here is likely related to *D. discoideum*’s defense against infectious microbes during phagocytosis [9, 52] rather than up-regulation of feeding in infected hosts compared to uninfected hosts. Notably, tricarboxylic acid (TCA) cycle and TCA metabolic process were down-regulated at 90 MPI but up-regulated at 360 MPI. The TCA cycle, glycolysis, and fatty acid metabolic processes are known subjects of metabolic programming by both host and symbiont during infection [53].

Down-regulated semantic clusters for 360 MPI were more diverse, with clusters for DNA replication and cell cycle. These terms likely reflect the specific timepoint we chose to ensure cells would have undergone at least once cell division after exposure to *P. bonniea*. In our pilot experiment, cell division occurred around 240 MPI and was detectable by a shift in cell size (forward scatter) during flow cytometry. Not present in the semantic analysis but worth noting from the enriched terms list were sphingoid and sphingolipid metabolic processes that continue to be downregulated from 90 to 2250 MPI. Sphingolipids are eukaryotic membrane lipids that are subject to hijacking by various pathogens [54, 55].

For a longer-term infection modeled by the 2250 MPI cells, signal transduction pathways for various transport functions were a significant cluster detected for up-regulated genes. Actin-filament based, post-Golgi vesicle, and endosomal transport as well as exocytosis was also present. For down-regulated genes, enriched GO terms included nucleoside and nucleotide metabolism, apoptosis regulation, and immune response and reactive oxygen species metabolism.

### Phagocytosis gene expression

We identified 63 specific genes involved in phagocytosis in *D. discoideum* [9, 56, 57] or its well-studied responses to pathogenic *Legionella pneumophila* and *Mycobacterium marinum* [58, 59] from the literature. Of these 63 genes, 40 were differentially expressed during our time series. We subsequently identified five clusters of phagocytosis genes based on patterns of gene expression changes over time (Fig. 5). Cluster A and B had maximum levels of gene expression at 360 MPI and are distinguished by whether transcript levels at 2250 MPI were ultimately higher (A) or lower (B) compared to negative controls.

Cluster A contained genes expected during all stages of phagocytosis, including phagocytic uptake regulator *racA*, vATPase subunit *vacA*, gram-negative lysozyme *alyL*, early trafficking genes including retromer *vsp35*, SNAREs (soluble NSF attachment protein receptors) *syn7A* and *syn8A*, and NADPH oxidase (NOX) 2 regulatory subunit *cybA* (p22^phox^). Post-lysosomal markers *vacB*, *vacC*, *mcln*, and autophagy receptor *p62* were also in this cluster. Cluster B contained more genes that are expected earlier during phagocytosis and related to F-actin polymerization during phagocytic uptake and early recycling of phagosomal membrane proteins. These included coronins *corA* and *corB*, a majority of Arp2/3 complex genes (*arcA*, *arcB*, *arcC*, *arcE*, *arpC*), endosome trafficking Rab GTPase *rab5A*, WASH complex genes (*wshA*, *ccdc53*), and *myoB* and *ehd*, both involved in early recycling.

In direct contrast to clusters A and B, cluster C showed a valley of transcript abundance at 360 HPI. These genes included the folic acid receptor *far1* that signals phagocytic uptake during feeding, and *clkB* that is also involved in uptake but specifically for gram-negative bacteria. Perhaps more interesting are *nramp1*, the iron transporter that is involved in nutritional immunity by exporting iron and manganese ions from phagosomes, and *ncfA*, the p67^phox^ that is required for activation of NOX2.

**Table 2:** Analysis of Deviance table for symbiont horizontal transmission in *D. discoideum* exposed to wildtype vs. ∆*tssH P. bonniea* mutants.

Variable	*X* ^2^	*df*	*P-value*	${\bf\eta}_{\bf{\textit p}}^{\bf{2}}$	*95% CI*
Infection prevalence	171.780	1	< 0.001	0.41	[0.26, 1]
Symbiont background	2.699	1	0.100	0	[0, 1]
Symbiont treatment	31.401	1	< 0.001	0.12	[0.02, 1]
Infection prevalence: background	13.458	1	< 0.001	0.02	[0, 1]
Infection prevalence: treatment	114.727	1	< 0.001	0.39	[0.23, 1]
Background: treatment	2.686	1	0.101	0.07	[0, 1]
3-way interaction	17.267	1	< 0.001	0.20	[0.07, 1]

**Table 3:** Analysis of Deviance table for symbiont horizontal transmission in *D. discoideum* exposed to wildtype, ∆*tssH*, and *ptssH P. bonniea* mutants.

Variable	*X* ^2^	*df*	*P-value*	${\bf\eta}_{\bf{\textit p}}^{\bf{2}}$	*95% CI*
Infection prevalence	755.277	1	< 0.001	0.68	[0.55, 1]
Symbiont background	4.153	1	0.042	0.02	[0, 1]
Symbiont treatment	12.575	2	0.002	0.03	[0, 1]
Infection prevalence: background	37.472	1	< 0.001	0.45	[0.27, 1]
Infection prevalence: treatment	12.502	2	0.002	0.19	[0.03, 1]
Background: treatment	1.566	2	0.457	0	[0, 1]

**Figure 4:**
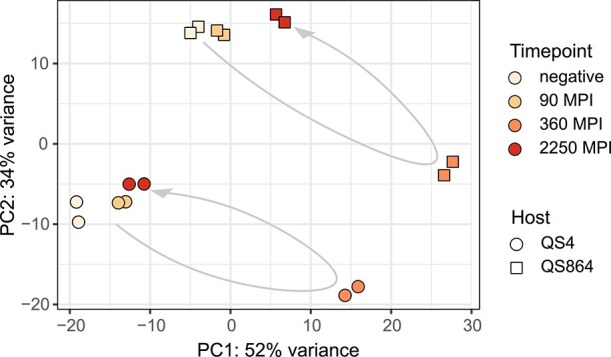
Overall gene expression patterns of *D. Discoideum* hosts infected with either wildtype or ∆*tssH P. Bonniea* strain bb433 are not significantly different from each other. Time series samples were collected at 90, 360, and at 2250 MPI. Manually drawn arrows show the trajectory of gene expression across timepoint within each host strain.

Cluster D genes showed consistent increase in transcript levels over our experimental time series. Several WASH complex genes were included here (*swip*, *fam21*, *washc5*), as well as genes associated with earlier stages of phagocytosis, *Dd5P4* (inositol 5-phosphatase 4) and large GTPase *dymA*. Cluster E genes showed consistent decrease in transcript levels over time. Only two genes were included but both are crucial for phagosome maturation as *rab7A* regulates the delivery of lysosomal hydrolases and *CP-p34* is the first lysosomal hydrolase that is delivered in *D. discoideum*.

## Discussion

### Function of T6SS in the symbiosis

The contractile injection system of the T6SS is a key factor for virulence in many gram-negative bacteria used to deliver effectors directly into eukaryotic host cell cytosols [3, 25]. By disrupting the *tssH* ATPase gene in the shared T6SS, we created a T6SS unable to retract its sheath for repeated firing [27]. By comparing wildtype vs. ∆*tssH* phenotypes, we characterized how a disrupted T6SS *tssH* affects *P. bonniea*’s ability to form a symbiotic relationship with *D. discoideum*. Increasing infection prevalence of *P. bonniea* had a negative effect on host fitness as previously observed [29]. However, the shared T6SS does not appear to directly affect *P. bonniea* virulence in regard to host fitness (Fig. 1). The lack of difference in host fitness outcomes was supported by a similar lack of differential gene expression between *D. discoideum* infected with wildtype vs. ∆*tssH P. bonniea* bb433. Instead, wildtype *P. bonniea* had significantly higher rates of horizontal transmission compared to ∆*tssH* (Fig. 3). The level of transmission we observed in ∆*tssH* mutants reflects the level of horizontal transmission possible with a single bout of T6SS firing and effector delivery in comparison to the wildtype where the T6SS would have been able to fire multiple times.

We also observed a significant difference in the range of infection prevalence achieved by wildtype vs. ∆*tssH* symbionts which was exacerbated over additional social stages (Fig. 2). The host fitness experiment incorporates one social stage which occurs once *D. discoideum* plated with *P. bonniea* depletes its food bacteria and forms fruiting bodies. Here amoebas are continuously able to gain symbionts from their petri dish environments and from other amoebas, presumably through either vertical or horizontal transmission. The symbiont transmission experiment incorporates two social stages because pre-infected amoeba spores have already experienced a social stage. These spores are grown without any additional *P. bonniea*, and during the experiment, pre-infected amoebas hatched from those spores are washed several times prior to mixing with uninfected amoebas on sterile nitrocellulose filters. Here uninfected amoebas gain symbionts primarily horizontally from pre-infected amoebas during the social stage that follows. A reduction in infection prevalence occurred for both wildtype and ∆*tssH* over the course of these two experiments with increasing numbers of social stages. This indicates that symbiont prevalence decreases in the absence of opportunities for environmental acquisition. The exacerbated difference in infection prevalence between wildtype vs. ∆*tssH* suggest that the shared T6SS significantly contributes to the ability of *P. bonniea* to stay associated with its hosts over multiple social stages, and ultimately over evolutionary time scales.

Bacteria use secretion systems of the same type for several different purposes. For example T6SS can be used against other bacteria, eukaryotes, or both [60]. However T6SS within the same genome tend to have distinct roles. For example, *Burkholderia glumae* BGR1 is a plant pathogen that possesses four T6SS [61]. Two of its T6SS act independently as virulence factors against its host plant rice, while a third T6SS is used in competition against other endophytic bacteria. Of the five T6SS possessed by *Burkholderia thailandensis*, T6SS-5 is the only T6SS that has an anti-eukaryotic role, while T6SS-1 has a cell contact-dependent anti-bacterial role [19]. The decreased infection prevalence observed in our experiments for ∆*tssH* (Fig. 2) could potentially be because the T6SS shared by these amoeba symbionts maintains or enhances the competitive ability of these bacteria against food bacteria in the lab environment. However, this potential role cannot explain why the rate of horizontal transmission is significantly lower for ∆*tssH* symbionts (Fig. 3). Therefore, a potential anti-eukaryotic role is more likely for the shared T6SS. This role may be related to proliferation within the host cell or transmission across host cells. For example, lower rates of transmission for ∆*tssH* may be because fewer symbionts cells are available for transmission or due to direct impairment of transmission. However if proliferation is affected by the shared T6SS we would expect to see some degree of difference in host response to wildtype vs. ∆*tssH* symbionts, either in the form of different slopes of host fitness outcomes (Fig. 1) or transcriptional differences (Fig. 4).

The known functions of T6SS in the host-pathogen context are determined by the delivered effectors that act to promote or prevent cytoskeletal rearrangement [3]. The shared T6SS is closely related to T6SS-5 of the *B. pseudomallei* complex that includes *B. thailandensis* [18]. Therefore we look to what is known from that system for insights into how *P. bonniea* transmission may be affected by the shared T6SS and for predicting the identities of key effectors. *B. pseudomallei* escapes the phagosome using its T3SS-3, then its two-component system VirAG senses glutathione within the host cytosol and activates its T6SS-5 [62]. The adjacent effector proteins VgrG-5 and TagD-5 localize to the bacterial poles and assist in host cell-to-cell membrane fusion, which enables pathogen transmission [63]. Our experiments show that the shared T6SS significantly affects symbiont horizontal transmission during social stages. Based on its similarity to T6SS-5, we predict that this secretion system directly impairs symbiont transmission rather than proliferation. We argue that successful horizontal transmission appears to be important for sustained symbiotic association, that must precede potential symbiont adaptation to the *D. discoideum* host environment. We previously found genomic patterns of host adaptation in the reduced genomes of *P. bonniea* and *P. hayleyella* [18], and *P. hayleyella* has higher abundance in soil microcosms when D. discoideum is present rather than absent (*P. bonniea* was not tested) [64].

**Figure 5:**
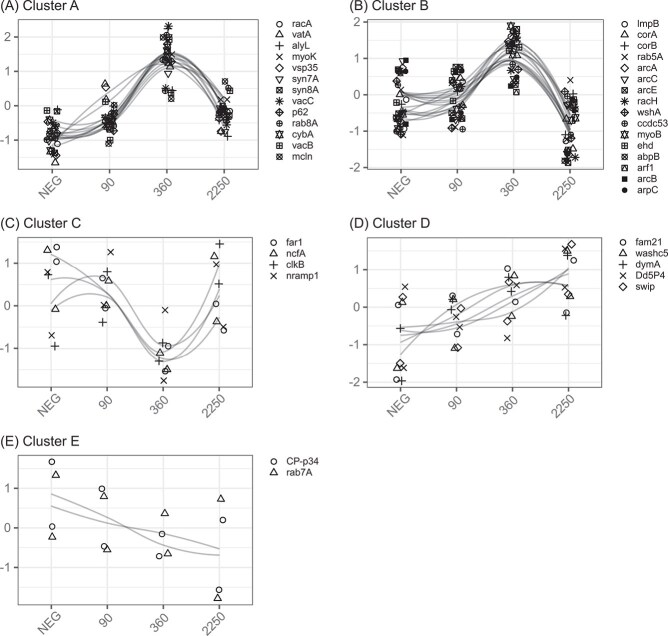
Differentially expressed phagocytosis genes formed five clusters (A–E) based on expression changes over our time series experiment. Normalized transcript abundance is shown over four timepoints: NEG (negative control), 90, 360, and 2250 MPI.

### Phagocytosis perturbation during symbiotic association

Although there were no differences in differential expression between wildtype vs. ∆*tssH*-induced host responses, our time series analysis of *D. discoideum* hosts offered an opportunity to better understand host-symbiont crosstalk during early stages of association. Our results suggest how *D. discoideum* may fail to phagocytose symbiotic *Paraburkholderia*. Phagocytosis in *D. discoideum* has many parallels to that in mammalian macrophages [9, 57]. Briefly, cell surface receptors binding to bacterial cell wall components trigger phagocytic uptake via F-actin polymerization driven by Arp2/3. Several proteins of the phagosomal membrane are subject to early recycling via WASH (WASP and SCAR homolog) and retromer complexes. Then delivery of vacuolar proton pump vATPase leads to acidification of the phagosome. Afterwards, lysosomal hydrolases are delivered and subsequently removed by endosomes. Levels of reactive oxygen species and metal ions are also manipulated by the host for successful killing. After nutrient extraction, what is left is called the post-lysosome and it has accumulated various membrane proteins over the course of phagocytosis. Post-lysosome contents are subject to exocytosis via F-actin polymerization driven by Arp2/3, which would take place after late membrane protein recycling via WASH and retromer complexes.

The balance of reactive oxygen and nitrogen species (oxidants) and antioxidants is important for hosts to be able to sustain oxidative bursts during pro-inflammatory responses and phagocytosis [65–68]. Cellular antioxidant enzymes include superoxide dismutases that break down superoxide, catalases that break down hydrogen peroxide, and thiol peroxidases that break down organic hydroperoxides. Detox via thiol peroxidases occur through the thioredoxin and glutathione systems, and thioredoxin reductase and glutathione reductase can restore each peroxidase to their reduced state by consuming NADPH [69, 70]. We observed consistent down-regulation of cellular antioxidant enzymes (superoxide dismutases, thioredoxin peroxidases, thioredoxin reductases, and a glutathione reductase) throughout our time series. This suggests an oxidant burst would be lacking in phagosomes containing *P. bonniea*. In addition, both ceramide anabolism and catabolism are downregulated during infection with *P. bonniea*, suggesting a role for sphingolipid metabolism in symbiosis. Sphingolipid pathway enzymes are known to regulate phagocytic uptake, phago-lysosome fusion, autophagy, and apoptosis [71].

The phagocytosis-related patterns of gene expression are consistent with *D. discoideum* hosts failing to phagocytose *Paraburkholderia* symbionts (Fig. 5). The specific phagocytic genes that are progressively down-regulated presented the strongest evidence, indicating a reduction of lysosomal hydrolase delivery to the phagosome over time. In addition, three genes belonging to phagocytosis gene clusters A and B stood out because they were previously known as targets of *Legionella* or *Mycobacterium*. *Legionella* is known to target *rab8A* (secretory Rab GTPase) and *aft1* (small GTPase) in the process of preventing the now *Legionella*-containing vacuole from progressing through phagocytosis [59]. The *Legionella*-containing vacuole ultimately becomes associated it with the endoplasmic reticulum instead. The other gene *racH* (small GTPase) is important for *Mycobacterium* cell-to-cell spread via its unique ejectosome [72, 73]. Future studies are needed to understand the specific roles of these proteins in our unique host-symbiont context.

## Conclusion

The persistent *D. discoideum* and *Paraburkholderia* symbiotic relationship allows us to investigate the effects of a shared T6SS as a key factor for symbiotic association. Our results indicate that, similar to T6SS-5 in the *B. pseudomallei* complex, the shared T6SS of *Paraburkholderia* symbionts of *D. discoideum* significantly affects symbiont horizontal transmission after host cell entry and does not affect host fitness directly. Successful horizontal transmission significantly contributes to sustained symbiotic association over multiple social stages in the absence of opportunities for environmental symbiont acquisition. Therefore, the shared T6SS may be necessary for a long-term evolutionary relationship between *D. discoideum* and its *Paraburkholderia* symbionts. The lack of direct T6SS impact on host fitness was reflected in our RNA-sequencing results. But our time series analysis of transcriptional changes during early symbiotic association points to intriguing avenues for future study, to better understand how *D. discoideum-Paraburkholderia* crosstalk ultimately results in conditions allowing for a long-term symbiotic relationship to evolve.

## Supplementary Material

tssH_supplement_figures_ycaf005

tssH_supplement_tables_ycaf005

## Data Availability

RNA-sequencing data (raw and processed) are available at NCBI GEO GSE276651. Additional data and code supporting this work can be accessed at: https://github.com/noh-lab/tssH.
