# Supplementary material for: Symbiotic T6SS affects horizontal transmission of Paraburkholderia bonniea among Dictyostelium discoideum amoeba hosts
Source: ISME Commun. 2025 Jan 14;5(1):ycaf005. doi: 10.1093/ismeco/ycaf005 (PMC11882306; doi:10.1093/ismeco/ycaf005)
Supplement: tssH_supplement_figures_ycaf005 [file tssh_supplement_figures_ycaf005.docx]

Supplemental Figures for: Symbiotic T6SS affects horizontal transmission of *Paraburkholderia bonniea* among *Dictyostelium discoideum* amoeba hosts

Anna Chen^1^, Rachel M. Covitz^1,2^, Abigail A. Folsom^1^, Xiangxi Mu^1^, Ronald F. Peck^1^, Suegene Noh^1*^

^1^ Biology Department, Colby College, 5717 Mayflower Hill, Waterville, ME, 04901, USA

^2^ School of Medicine, University of Pittsburgh, 3550 Terrace Street, Pittsburgh, PA, 15213, USA

^*^ Corresponding author: suegene.noh@colby.edu

Supplemental Figure S1. Addition of a *tssH* expression plasmid into ∆*tssH* mutants (p*tssH*) rescues the symbiont transmission phenotype. Across both symbiont backgrounds, mean infection prevalence and horizontal transmission rate slopes were not significantly different between wildtype and p*tssH* rescues.

Supplemental Figure S2. Of the time series samples collected at (a) 90, (b) 360, and (c) 2250 MPI, more genes were differentially expressed by *P. bonniea*-infected *D. discoideum* hosts at 360 MPI relative to other timepoints*.* Genes that met false discovery rate thresholds of 0.05 (red) and 0.1 (green) for differential expression are indicated.

Supplemental Figure S3. Comparison of (a) up-regulated and (b) down-regulated differentially expressed *D. discoideum* host genes at each timepoint indicated the highest overlap between 360 and 2250 MPI than between other timepoints.

Supplemental Figure S4. Clusters of related terms identified through semantic analysis of enriched GO-terms for differentially expressed genes at each timepoint
